# A Report of Cases Treated at the Louisville Marine Hospital

**Published:** 1842-01

**Authors:** Richard W. Ferguson

**Affiliations:** Louisville


### THE WESTERN JOURNAL OF MEDICINE AND SURGERY.

JANUARY, 1842.

Art. I.—*A Report of cases treated at the Louisville Marine Hospital.* By Richard W. Ferguson, M. D.

In compliance with the requisitions of the Louisville District Medical Association, the committee on Autopsies beg leave to submit the following cases (all of which have occurred in public practice) as their report.

Upon the assumption of the duties of one of your committee at the Louisville Marine Hospital, commencing the fourth day of July, the following brief history of the patient, who is the subject of the first report, was given by the student in attendance, who had been particular in gaining information of his friends, at the time of his admission.

### Case I.

Henry Schneider, a native of Germany, by trade a tailor, and a man of temperate habits, was admitted on the 23d of June, 1841. His health, previous to this attack, had been generally good; but on the 15th of the month, eight days prior to his admission, he was attacked with pain in the limbs, back and head, with inability to protrude the tongue. These symptoms were soon followed by vomiting of bilious matter, loss of appetite, and high febrile excitement. He had been under the medical treatment of a German doctor, in the city, without any benefit or amelioration of symptoms. At the period of admission to the hospital he did not complain of much pain, and this was confined to the region of the liver. Pressure with the hand did not cause pain in any other part of the abdomen. His tongue was coated with a thick brown fur, edge and tip red, and perfectly dry. Some difficulty still existed in the protrusion of that organ, and its retraction seemed difficult and slow. Pulse 125 in the minute, full and resisting; skin hot and dry, thirst great.

Treatment—About twelve ounces of blood was taken from the arm, after which calomel, gr. xx., and ipecacuanha, gr. j. combined, were given every two hours: the spt. mindereri and sponging the surface of the body with tepid vinegar and water were used in conjunction, in order to moderate his fever. Under this treatment he seemed to mend. The evacuations, which were dark, offensive and thin, at first, in a few days assumed a more natural appearance.

The above mentioned treatment was pursued till within three days of the time he fell into my hands; when it was thought by his attendants that the specific action of the mercury had been obtained, and it was accordingly abandoned, substituting for it rhubarb and magnesia, which he was taking at the time he was transferred.

After carefully inspecting the condition of his mouth, a different belief was entertained, and a cautious exhibition of the mercury and ipecac was resumed. His tongue, at this time, (4th of July,) was red, dry in the centre, edge pasty, and still exhibited the brown coat; pulse, 100 in the minute; was free from pain and uneasiness in every part of the body; his mind was clear; skin dry.

A large crop of small white vesicles was discovered to occupy, without tenderness, his breast and abdominal parietes. The dose of calomel, commenced with, was ten grains with two of ipecacuanha, to be given every four hours with alternate doses of rhubarb, aloes, and jalap. The spt. mindereri was employed in conjunction with the above named purgatives, during the continuance of fever, and until free bilious discharges could be obtained.

July 5th. Patient has had several dark green evacuations, possessing a cabbage smell; his general condition about the same. Treatment continued.

6th. Patient rested well last night; complains of thirst; can cause pain by pressure upon the liver, far round toward the vertebral column; tongue unchanged; pulse 100 and soft; has had four dejections from the bowels, presenting the same colour and smell, and about the consistence of thick gruel; temperature of the body irregular. Prescription: Calomel, ℈j., camphor gr. ij., ipecac gr. ij. conjoined with spir. mindereri as before. Farinaceous articles of food, and animal broths allowed.

7th. Patient has rested well; is free now from pain; tongue somewhat improved; pulse 104 and more open and strong; skin soft and silky. He complains of loss of appetite, and of occasional thirst. He has had five or six discharges of blood from the bowels, which, in the aggregate, was estimated at half a gallon. This blood seemed to be about the consistence of thick gruel, dark and grainy, with some few coagula intermixed. Prescription: calomel ℈ij. every six hours.

8th. Rested well; no pain; feet and hands, this morning, rather cool; those white vesicles, already noticed, still continue on the body; thirst is moderate; tongue softer and cleaner; had five operations from the bowels in 24 hours; hemorrhage checked; is now passing off dark green cabbage-scented evacuations. Treatment pursued.

9th. Has had another good night; has no pain at any point; condition of his tongue unaltered, with the exception that its edge seems thickened, but no mercurial effects exhibit themselves; vesicles are disappearing from the upper part of the body, but are full and abundant about the groins and upper half of the thighs, intermixed upon the abdomen with small red splotches; his feet are cool; pulse 112 and moderately strong. Treatment continued.

10th. Patient did not rest well last night; is somewhat delerious this morning; tongue has become dry and adhesive; skin, nevertheless, is soft; pulse 106, and not so open and free as yesterday. Vesicles and petechiæ still conspicuous; has had four or five evacuations with a strong tendency to hemorrhage again. Prescription: Calomel ℈j. every five hours, until the above tendency subsides; beef tea ordered for nourishment.

11th. Tongue is less red but still dry; has thirst; pulse 104; skin very good; has had several dark green passages, with a slight admixture of blood and very offensive. Prescription: a dose of rhubarb and magnesia, ordered to-day to carry off the numerous and large doses of calomel he had been taking.

12th. Not so well; is weaker; breathing laborious; skin moist and clammy; tongue same; has had three operations, the consistence of thick gruel, with a slight tinge of blood, possessing, as before, the smell of cabbage; pulse 112, small and quick. Treatment continued.

13th. Patient decidedly worse; tongue soft on its edge, centre brown and rather dry; pulse 112; complains of being weak; sleeps with his eyes half open, the balls inverted; lies drawn up in bed, as one having a chill. Prescription: rest and nourishment. He died between the hours of 12 and 1 o'clock in the night.

Post-mortem held fifteen hours after death. Emaciation moderate; incurvation and rigidity of the fingers of both hands considerable.

The brain was given to Dr. Symmes, the Phrenologist, to exhibit and demonstrate to his class. The doctor stated, that it was not until he was preparing the brain for the usual hardening process, that he discovered considerable softening of the organ, and this was unusual at two points—making up the phrenological compartments of hope, and—

The whole substance of the brain had been so far altered from its normal state, as to render him apprehensive that entire satisfaction in witnessing its fibrous structure (which the doctor was anxious to unfold) could not, upon that occcasion, be given. Even in this softened state, the demonstation was sufficiently clear to show its fibrous structure; it being much more conspicuous, however, in some portions than in others. The ventricles of course were not examined.

It may be proper, just here, to remark, that the doctor found out the boarding house of this man, and was duly informed by persons who knew him, who stated that his habits and character were in strict conformity with the diseased appearances as revealed in the cerebral mass.

Lungs.—Old adhesions about the root of the right lung, which organ was otherwise healthy.

Heart—Rather smaller than natural, walls thin and flaccid.

Spleen—Small but healthy.

Liver—Of the natural size, but pale and remarkably indurated; about the centre of the organ, an irregular yellowish discoloration, about four inches in diameter, gradually fading into the surrounding paler structure. Upon cutting into the organ it presented somewhat a carnified appearance.

Gall Bladder—Distended with dark green thin bile.

The stomach and the whole of the intestinal tube healthy, until we came to the ilium when it was discovered that very considerable ulceration had taken place; the blood vessels going to these ulcers were very much engorged, but there was no appearance of blood upon their surfaces. The ulcers increased in size, depth, and frequency down to the commencement of the colon, and in the caput coli there were two small ulcers.

Kidnies—healthy in their structure. The right had a vesicle upon its outer surface, which might embed the half of a common size marble, filled with serum.

The mesenteric glands were found considerably enlarged. Pancreas, healthy.

### Case II.

Domineck Foissel, a native of France, a man who had been once intemperate in his habits, was seized with diabetes, upwards of three years ago, and was admitted into the Louisville Marine Hospital on the 21st of June, 1841. The disease, he said, came on gradually, but could not trace or refer it to any particular cause. His health, previous to this, had been always good, excepting once, when he had asthma, of which he had been cured. The diuresis has increased upon him. At the time of his admission he passed, in the 24 hours, eighteen pints of very clear, pale urine; the discharges from the bowels were involuntary; tongue perfectly clean; skin moist; towards evening he had slight exacerbations of fever. The treatment which was adopted in the absence of the attending physician for two or three days, was one which had for its chief object, the restriction of the diuresis; he was accordingly put upon a combination of bark, uva ursi, and opium, which had a good effect, and was afterwards continued with the addition of the precipitated carbonate of iron, of which xxv. gr. were given three times a day. All farinaceous articles of food, all vegetables, excepting cabbage, were forbidden. In a few days the first prescription was discontinued, and quinine and opium were substituted. This forms a brief outline of the treatment up to the time he fell into my hands, upon the 4th of July. The following were the symptoms detailed by him to me, interrogatories being previously made.

Appetite has been always good, and his stomach easy after meals; thirst great, but less of this latterly; mouth dry when quiet, becomes moist when he talks; tongue smooth and clean; taste varies, being either salt, sweet or bitter, and never good; skin, when he was first taken, felt rough and dry, is now soft and pleasant; emaciation, loss of strength and weariness considerable; aversion to exercise—this has to be forced on him; has always more or less pain, with sensation of weakness in the region of the kidnies. The state of his bowels for the first month was a constipated one, since then they have become much more relaxed; loss of virility; lower half of his body and feet cold; his legs œdematous, at one time even as high as the knees. The swelling is now confined to his ankles, and is very slight; acid eructations have come on lately, but are not troublesome; flatulency considerable; eyes always clear, vision much diminished, but has neither vertigo nor headache. Dyspnœa he had previous to this attack, at the time he was laboring under asthma, no difficulty in breathing since. His gums looked ulcerated, as persons having a scorbutic affection; mind disposed to be cross and irritable.

He was working on a farm when this disease came gradually upon him, accompanied with pain in the back. He does not believe that any other member of his family ever had the like disease.

July 4th. His bowels at this time were frequently operated on, and were small and discolored, probably by his ferruginous medicines. Treatment continued.

5th. Bowels less operated on; tongue as usual, perfectly smooth and clean; quantity of urine the same; his evacuations were similar to those already described.

6th. Quantity of urine the same; condition of the bowels unchanged: increase of opium to j¼ gr. at each dose, taken thrice daily.

7th. The state of the patient the same, prescription as heretofore.

8th. Not so stupid and dull this morning as he was yesterday; this state was possibly produced by the anodyne; has some pain in the back. Prescription: lactucarium gr j., Dover's powder gr. v. combined, and given three times a day.

9th. Feels pretty well this morning; diuresis reduced to 8½ pints; tongue unaltered. Prescription continued.

10th. Patient has had nine small discharges from his bowels since yesterday, without any straining; diuresis not quite 8 pints. Prescription continued.

12th. Diuresis 8½ pints; complains of alum whey producing flatulency; condition of his bowels unaltered; no pain whatever about the abdomen. Prescription: ol. anisi gtt. ½, two or three times a day until relieved of flatulency and to suspend the alum whey.

13th. Is relieved of flatulency; diuresis reduced to six pints. Prescription, rest.

14th. Has had nine small dark-looking discharges as usual, diuresis reduced to seven pints, this depends greatly upon the amount of water taken during the day; with a view of restricting him the more, his drinks are ordered to be tepid.

15th. Has been operated on ten times since yesterday's visit; the last evacuation looked more natural; has some uneasiness in his bowels; flow of urine reduced to 6¾ pints; has been using cabbage at dinner. Prescription: quinine gr. j., opium gr. j. combined, to be given three times a day and to withdraw his cabbage.

16th. Feels easier in his bowels; they are less operated on; passages still small and in colour like soft soap. Prescription: lactucarium and Dover's powder as given on the 8th instant.

17th. Patient does not feel so well; recurrence of purging; stools the same in character; tongue still clear; pulse, which has been generally natural, is now 96 in the minute, regular and quick; complains of pain in the region of the liver, with loss of appetite. Prescription: hydrarg, cum creta gr. v., opium gr. ½ combined to be given every four hours. Decoction of rad. columba or quassia, to be used as a tonic. Tinct. opium gtt. x. with camphor water alternately.

18th. Patient has had no discharge of urine since 12 o'clock, the night of the 16th inst. At that time he passed about ½ pint; is free from pain; did not perspire yesterday; this morning the skin acts; diarrhœa urgent all day yesterday, at intervals of fifteen or twenty minutes. The dejections looked like yeast suspended in a thin whey-colored fluid; complains of anorexia; thirst quite moderate now; tongue unaltered; pulse, in a recumbent posture 70, sitting up is 76 in the minute. Prescription: hydrarg. cum creta gr. v. g. opium gr. j., four times a day.

19th. Diuresis, two-thirds of a pint yesterday morning, (just two days.) He has had no pain, no distension about the hypogastric region. Discharges from the bowels copius, frequent and involuntary. There is more redness than usual at the tip of the tongue; urine has a natural smell and color. Prescription: rad. columba gr. v. g., opium gr. j. to be given; the mercurial preparation discontinued.

20th. Rested well; feels stronger and better this morning; discharge of urine in 24 hours not quite one pint; tongue clean, particularly its edge, which has likewise a smoother appearance than the centre; complains of some pain in the back; pulse 70; evacuations still involuntary, and runs from him continually in a small stream. Prescription: add kino or the catechu to the powder of yesterday, and apply a burgundy pitch plaster to the small of his back.

21st. Rested very well last night; feels better this morning. Prescription the same.

22d. Rested well; feels stronger and better; diuresis one pint and a half since yesterday at 10 o'clock; head swims when he sits up; appetite good; thirst moderate; is not so much purged, but when upon the vessel he remains there upwards of an hour; passages are a pale yellow color, and of the consistence of soft soap. Prescription: give one grain of quinine three times a day, alternating with powders composed of opium and uva ursi.

23d. Somnolency very great this morning; diuresis to two pints in 24 hours; has had two dejections of a pale yellow color, and about the consistence of soft soap. Prescription: omit the uva ursi.

24th. Tongue somewhat pasty, edge soft; pulse 58 in the minute; back is a little better; diuresis not quite a pint, but his bowels have been operated on largely since midnight; stools thin and whey-like. Prescription: calomel gr. ij. g., opium gr. j. mixed, and to be given every four hours.

25th. Bowels still very much relaxed; discharges are involuntary again, and are thin and watery, having very much the color and smell of sour whey. The meat he eats passes off unchanged. Prescription: calomel gr. ij. opium iss., every four hours; his nourishment to be formed of gruel made out of parched rice; sinapisms to lumbar region.

26th. Rested pretty well in the latter part of the night; has been less purged; some little œdema about the ankles, more or less of this has occasionally presented itself throughout the attack; discharge of urine twice, half a pint each time; his bowels have been moved five times since yesterday, about the consistence of thick gruel, color a pale yellowish green; pulse 56. He expressed a desire for a little mush with salt, which was allowed.

27th. Rested well last night, though his bowels were occasionally much operated on; stools thin, and indicated feeble powers of digestion; pulse 56. Prescription: rhubarb, opium, and kino 2 gr. each, with quinine gr. j. to be given alternately with the preceding, in intervals of four hours; sinapisms to the back.

28th. Bowels in the same state; voided a pint of clear whiskey-colored urine; ankles a little swelled.

Died between the hours of three and four, on the 29th of July without any person knowing it. The nurse had been in attendance upon him at three o'clock when he was troubled still with this looseness of the bowels, but did not complain of any thing beyond this.

Post-mortem examination instituted about ten hours after death, when the following appearances were revealed:

Emaciation.—Moderate; very remarkable flexibility of the superior extremities with the usual stiffness of the inferior. The left ankle somewhat œdematous; strabismus of left eye, considered however to be accidental, there was no unnatural state of the pupils. As it was thought by your committee that the whole force of diseased action had been concentrated upon the abdominal viscera, and that it was here that pathological inquiries might be extensively made; the head and thorax were consequently omitted in the examination; nor was their belief unsupported by the morbid appearances upon opening this cavity, which were as follows:

Liver—Somewhat enlarged; its external and internal appearance pretty good, but it possessed an uncommon degree of firmness. The only part which could be at all broken into by a thrust or continued pressure with the finger, was the superior thick portion of the right lobe, the edge or margin of whichwas likewise somewhat thickened.

Gall bladder—Moderately filled with very thick black bile.

Spleen—Small but healthy.

Stomach—Its whole surface was thickly covered with mucus. The mucous membrane itself was found considerably thickened, and very firm, so much so as to enable you to tear it off in strips. There was no other alteration than general thickening of this membrane throughout the intestinal tube, until arriving at the colon. The surface of this bowel was copiously studded over with vesicular looking pimples, which, however, contained matter of more decided consistence than is to be found in vesicular bodies, for it seemed somewhat caseous. The cœcum, was found bound down by strong adhesions to the whole iliac fossa. Within the sigmoid flexure of the colon were presented one or more singular bodies bearing the appearance of warty excrescences of a dull mulberry hue. The largest of these was not unlike in form and size the kidney bean; rather thicker and rounder than this, and pendulous from the mucous coat. These strange vegetations were more abundant, or rather more matured in the rectum.

Here they were found in different stages of perfection, ranging from the hard granulation, to the more ensanguined or purplish bodies already mentioned. These, at length, became more and more elongated in figure, and looked like numerous teats, or rather a collection of fungi, measuring from a fourth to half an inch in length. Each mushroom growth had, for its cap, a suet-like deposition, which could be easily removed. There were likewise discovered one or more surfaces or annular marks, between the size of a large spangle and a half dime, regarded by your committee as vestiges of the dugs or fungi already described, for they correspond to their bases and were possibly removed by absorption, leaving these smooth plates within the rings, whose slightly thickened edges formed the only landmarks of prior disease at these points. The whole alimentary canal was thickly coated with a secretion of mucus.

Kidneys—Broader, thicker, and more rounded in form; somewhat hard to the touch, and rather more of a pink color than in the sound state. Their pelves, likewise, partook slightly of this hue. The investing cellular membranes and fatty portions surrounding these, and also other organs seemed to have been changed into a gelatinous amber-colored substance, which was found to be of a lighter color about the omentum. There was no remarkable odor perceptible about the urinary organs.

Bladder—Very much distended with insipid urine, which possessed also a brandy colour; mucous membrane here, as well as elsewhere, much thickened.

Mesenteric glands—Enlarged.

A small quantity of the whey-like discharges from the bowels was tested both by the action of heat and by a solution of corrosive sublimate in distilled water. Heat did not coagulate it, the solution changed its color and condition instantly, rendering it pinkish and flocculent, was at first suspended and floated in the solution, but in a little time it subsided to the bottom of the phial. The like quantity of urine (his last discharge) was treated in a similar manner, without coagulating by heat, and yielded a pale yellow deposite. This urine was perfectly clear, very much in colour like whiskey, and pricked the tongue as if there was something alkaline in its combination.

Different specimens of urine with, and without the tests exhibited.

In closing this observation, it may be proper to remark that very little was done, after he became my patient with the view of effecting a cure. Such a termination, however desirable, was considered, under existing circumstances, impossible. The long continuance of the disease, its formidable nature, the inefficiency of art to combat it upon any principles of cure that were adopted by my predecessors, and that have been suggested by the profession, were quite sufficient to convince me that very little benefit, either by medicines or regimen, even in the way of alleviation could be obtained in the case. How far the above supposition was correct, the history of the patient up to this period induced it, the lesions revealed after death confirmed it. Nor was there any thing discovered in this examination, whereupon to base any certain conclusions respecting the nature of the disease.

All that can be said is, that if the kidneys, the disordered functions of which, and the pain located in the lumbar regions, conferring the characteristic features of the disease persuade to the opinion that these organs were the primary seat of it; the disordered state of the bowels, even prior to his admission to the hospital, and its unceasing concomitancy after this, together with the sudden and easy metastasis from the urinary to these organs shows that the chylopoietic and assistant chylopoietic viscera generally were not now in a less deranged state.

### Case III.

Jas. B. Read, æt 36, a native of New York, was put ashore at Portland, having arrived there in some steamboat, and was brought to the Louisville Marine Hospital on the 4th of August. He was totally insensible at the time of admission, from which state he could not, for an instant, be roused, nor could any very satisfactory account be given of him; reported, however, as having been troubled with diarrhœa.

His bowels were moved the day he became an inmate of the institution. The dejections, contrary to what was anticipated from the report given, were noticed to be neither thin nor frequent.

August 5th. Patient still insensible; lies mostly with his body bent; knees drawn upwards towards his breast; occasional jactitation, and frequent low moaning, as one in extreme pain. Tongue, as seen through his open mouth, thickened, dry, and red with a brown coating in the centre; skin cool, and remarkably lifeless or inelastic; pulse very small and quick; tenderness and some fulness about the region of the liver.

Blisters and sinapisms had been applied previously to my seeing him. The latter were ordered to be re-applied to his arms, and the ammonia julap to be given every half hour or hour until reaction was established. This could not be effected and he died at 7 o'clock, P. M.—insensible to the last.

Post-mortem appearances twenty hours after death. Emaciation moderate; considerable incurvation and rigidity of fingers.

Brain—Serious effusion thrown out upon the arachnoid membrane, with a heavy coating of lymph deposited on the anterior lobes; at the disparting of the optic nerves, a small coagulum of blood, was found lying near the left nerve.

The ventricles were not examined in consequence of a disposition expressed upon the part of one of your committee to save it entire for the Medical Institute, as a fine specimen of arachnitis, which was most cordially allowed.

Liver—Somewhat enlarged; its general surface possessed a turmeric color with one or more lighter yellow spots about the centre of the right lobe, the left had its margin similarly discoloured, and extending nearly all around toward the great fissure, and another at this point. The superior margin of the right lobe differed again from any other portion; its color being a light pink; the whole organ possessed somewhat an unctuous feel, was regarded therefore by two of your committee, who made the examination, as coming under the appellation of "fatty degeneration of the liver."

Gall bladder—Distended with thick dark bile; cutting into it, thick particles flowed off not unlike softened tea leaves.

In the absence of morbid specimens, which were unfortunately lost, an attempt has been made by paintings to supply this want.

Louisville, November, 1841.

